# Prevalence of malaria infection in Butajira area, south-central Ethiopia

**DOI:** 10.1186/1475-2875-11-84

**Published:** 2012-03-23

**Authors:** Adugna Woyessa, Wakgari Deressa, Ahmed Ali, Bernt Lindtjørn

**Affiliations:** 1 Ethiopian Health and Nutrition Research Institute, P.O. Box 1242, Addis Ababa, Ethiopia; 2 School of Public Health, College of Health Sciences, Addis Ababa University, P.O. Box 9086, Addis Ababa, Ethiopia; 3 Centre for International Health, University of Bergen, Bergen 5021, Norway

**Keywords:** Malaria prevalence, Cross-sectional survey, Highland malaria, Ethiopia

## Abstract

**Background:**

In 2005, the Ethiopian government launched a massive expansion of the malaria prevention and control programme. The programme was aimed mainly at the reduction of malaria in populations living below 2,000 m above sea level. Global warming has been implicated in the increase in the prevalence of malaria in the highlands. However, there is still a paucity of information on the occurrence of malaria at higher altitudes. The objective of this study was to estimate malaria prevalence in highland areas of south-central Ethiopia, designated as the Butajira area.

**Methods:**

Using a multi-stage sampling technique, 750 households were selected. All consenting family members were examined for malaria parasites in thick and thin blood smears. The assessment was repeated six times for two years (October 2008 to June 2010).

**Results:**

In total, 19,207 persons were examined in the six surveys. From those tested, 178 slides were positive for malaria, of which 154 (86.5%) were positive for *Plasmodium vivax *and 22 (12.4%) for *Plasmodium falciparum*; the remaining two (1.1%) showed mixed infections of *Plasmodium falciparum *and *Plasmodium vivax*. The incidence of malaria was higher after the main rainy season, both in lower lying and in highland areas. The incidence in the highlands was low and similar for all age groups, whereas in the lowlands, malaria occurred mostly in those of one to nine years of age.

**Conclusion:**

This study documented a low prevalence of malaria that varied with season and altitudinal zone in a highland-fringe area of Ethiopia. Most of the malaria infections were attributable to *Plasmodium vivax*.

## Background

In most parts of Ethiopia, malaria is unstable and seasonal because of the altitude and climatic factors [1]. Areas at altitudes between 1,600 and 2,000 m above sea level (masl) are epidemic-prone hypoendemic zones of malaria [2]. Malaria epidemics are thought to occur among populations settling as high as 2,500 masl [3]. Thus, people in the Ethiopian highlands, which were considered previously to be malaria free, are at an increasing risk of malaria [4]. The prevalence of malaria has been found to vary among locations [1,2], possibly because of the country's heterogeneous topography, and weather variables [5].

In Ethiopia, *Plasmodium falciparum *and *Plasmodium vivax *account for 60-70% and 30-40% of malaria cases, respectively [6]. *Plasmodium falciparum *has been the major cause of epidemics, and of most malaria deaths [7]. *Anopheles arabiensis *is the main vector of malaria, and it has a wide geographical distribution. *Anopheles pharoensis, Anopheles funestus *and *Anopheles nili *are secondary vectors in some areas [6]. Ethiopia's Ministry of Health has been successful in reducing malaria prevalence, and the number of villages affected by malaria epidemics decreased from 681 in 2004 to three in 2008 [8]. Unfortunately, malaria remains among the leading causes of outpatient visits and hospital admissions in the country [9].

There is a paucity of information on the prevalence of malaria in the Ethiopian highlands, but such information is considered vital to focus and improve malaria interventions. In addition, estimates of malaria prevalence could be used in the development of early-warning mechanisms for malaria. Previous malaria prevalence studies have been performed either at lower altitudes in areas of high transmission [10,11], or in highland urban settings [12,13]. Furthermore, all of them were conducted during seasons of peak malaria transmission. This study was designed to estimate the prevalence of malaria by taking temporal and spatial variation into account. Temporal variation of malaria was addressed by performing repeated cross-sectional surveys of malaria in three distinct seasons of each year for two consecutive years. To address the spatial variation, villages at different altitudes lying between 1,800 and 2,300 masl were selected randomly and investigated. This study provides information on the community-based prevalence of malaria in epidemic-prone highland areas in south-central Ethiopia using active survey data.

## Methods

This study was part of the Ethiopian Malaria Prediction System, a multi-disciplinary research project designed to improve early warning systems for malaria in Ethiopia [14]. This study involved repeated population-based surveys to estimate the seasonal prevalence of *Plasmodium vivax *and *Plasmodium falciparum*.

## Population-based surveys

### The study area and population

**Figure 1:**
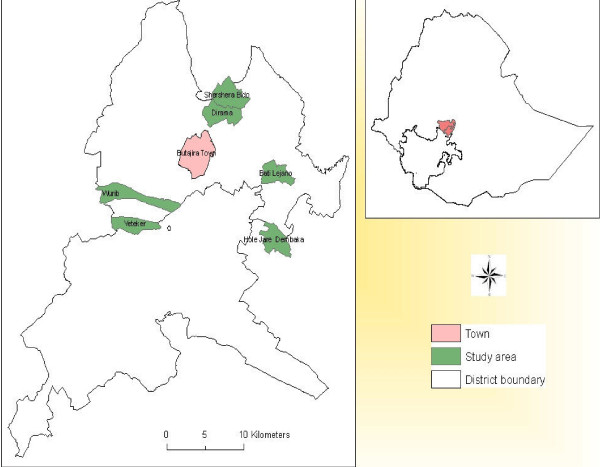
**Location of the study area, Butajira area, South-central Ethiopia**.

This study was conducted in six kebeles (the smallest administrative units) located about 130 km south of Addis Ababa in the Meskan and Mareko districts of the Gurage Zone in Ethiopia (Figure 1), which is designated as the Butajira area. These six kebeles are the location of the Demographic Surveillance Site (DSS) of the Butajira Rural Health Program (BRHP) [15].

There were 58,335 people living in the BRHP DSS in 2008. Half (50.1%, n = 29,243) of the population were female. Of the total population, 46% lived in the study area. The study area is part of an altitudinal transect between 1,800 and 2,300 masl. The mean annual rainfall in the study area during the last nine years was 945 mm (yearly range 510 mm to 1,329 mm). However, annual rainfall was below average in 2009 and 2010. The main rainy season usually occurs from June to September. The mean temperature was 18.2°C, with average annual minimum and maximum temperatures of 10.0°C and 26.3°C, respectively.

Most people in the area practise subsistence farming. The main cash crops are pepper at low altitude and *khat *(*Catha edulis*) in high altitude areas. The main staple food in the highlands is Enset (*Ensete ventricosum*), and in the lowlands maize, wheat, barley and *Teff *(*Eragrostis tef*) are consumed. Animal husbandry is also performed on the farms. The Odamo, Kelakel and Asass rivers flow through the area. The population usually seeks health services at the health posts found in each kebele, three health centres (two at low and one at high altitude) and two hospitals in the area.

### Sample size calculation

Estimation of the sample size for the assessment of malaria prevalence was based on a 4.1% prevalence reported from three Ethiopian regions [16]. Employing assumptions of expected prevalence = 4%, margin of error = 1%, α = 5% (95% confidence level), design effect = 2 and 15% non-response rate, a sample size of 3,393 persons was calculated. Thus, with the assumption of an average family size of 4.5, a total of 3,393 people were recruited from 750 households. The sample size was calculated as n = (1.96)^2 ^(0.04) (0.96)/0.01)^2 ^= 1,475; total n = (1,472 × 2) + (1,475 × 2 × 0.15) = 2,950 + 443 = 3,393; and 3,393/4.5 = 750.

### Survey design and sampling

The study used a community-based prospective design based on repeated cross-sectional surveys. The surveys used a multi-stage random sample design with kebeles as the first-stage unit, villages as the second, and households as the third stage [17].

Six rural kebeles were selected (Hobe, Bati Lejano, Dirama, Shershera Bido, Yeteker and Wurib), with two areas each representing low altitude (1,800-1,899 masl), mid-level (1,900- 1,999 masl) and high altitude (2,000-2,300 masl). The targeted households residing in those kebeles numbered 4,816. Sixteen villages with 750 households were selected randomly from the six kebeles, using probability proportion to size (PPS) sampling. Given that a lower prevalence of malaria was expected in the highlands, more villages and households were selected proportionally. Altitude readings for the households were recorded using a hand-held Global Positioning System (Garmin eTrex^®^).

### Prospective data

Six cross-sectional surveys were performed on the same households for two consecutive years. The surveys were conducted in October-November 2008 (a month after the main rainy season), January-February 2009 (dry season), June-July 2009 (main rainy season), October- November 2009, January-February 2010, and June 2010. To ensure maximum participation, households with absentees were re-visited once.

Thin and thick blood smears were collected from all family members who consented to the study. Blood specimen processing, examination, and reporting of results were performed using standard guidelines [18]. In brief, the smears were air dried, placed in slide boxes, and examined by a trained microscopist at the field laboratory in Butajira. Thin films were fixed with methanol, and both thin and thick films were stained with 3% Giemsa stain for 30 min. Microscopic examination was done at 1,000× magnification. During the microscopic examination, a slide was regarded as negative after 100 fields had been examined without finding any parasites. Gametocytes were counted against 1,000 leukocytes.

To assure quality of the microscopic examinations, all positive and 10% of the negative slides were re-examined by a second microscopist. In the six surveys, seven of 2,094 slides (0.3%) showed discordant results. The agreement between the first two readers was good (kappa = 0.88). A third reader, blinded to the previous results, re-examined the seven discrepant slides (six vivax malaria and one negative).

### Data management and statistical analysis

A training manual was prepared for the data collectors, who were trained in data collection. Data collection was performed using a pre-tested structured questionnaire and format prepared for this purpose. All data collectors were fluent in the local spoken languages. Data collection in the house-to-house survey was guided by DSS enumerators or the supervisors of each study kebele.

The questionnaire was translated from English to the local language (Amharic) and back to English to check for consistency. All family members who consented to participate in the study were included. The principal investigator and a supervisor from Butajira DSS performed the daily field supervision and cross-checking of the completed questionnaires.

Data entry and cleaning were performed using Epi Info version 6 (Centers for Disease Control and Prevention (CDC), Atlanta, Georgia (USA)). The data were analysed using SPSS version 18.0 (*SPSS*, Inc., 2009, Chicago). Descriptive statistics were applied to examine the characteristics of the sample; 95% confidence intervals were also calculated. Malaria prevalence was computed by dividing the number of people who showed infection with *Plasmodia *species by the total number of people examined from the study population. Households with missing values were not considered for analysis.

### Ethical considerations

The study received ethical clearance from the Institutional Review Board of the College of Health Sciences of the Addis Ababa University, and from the Ministry of Science and Technology of Ethiopia. The local and regional health authorities were informed. Individual informed consent was obtained from adults, and from the parents or guardians of children aged less than 18 years. Blood specimens were collected aseptically using an alcohol swab and disposable blood lancets by trained staff. All people found to be malaria positive during the survey were treated directly according to the national guideline [19]. Febrile patients were screened for malaria parasites on the spot using malaria RDTs (CareStart^®^), and blood slides were also collected for the survey to investigate the nature of the malaria parasite using light microscopy. Malaria positive cases detected using malaria RDTs were treated immediately. Moreover, those found to be malaria positive on Giemsa-stained light microscopy were also treated. *Plasmodium vivax *positives were treated with chloroquine, 25 mg/kg for three days (10 mg base per kg on days 1 and 2, and 5 mg base per kg on day 3). Artemether-lumefantrine (20 mg artemether plus 120 mg lumefantrine in a fixed dose combination) was administered, based on body weight, two times a day for three days to *Plasmodium falciparum*-positive patients. Moreover, Coartem^® ^and chloroquine stocks were kept at the health posts for treatment of cases that occurred between our repeated surveys.

## Results

### Characteristics of the study participants

In the six surveys, 19,207 blood slides were collected during house-to-house visits to 738 households in six kebeles, with an average of 3,780 persons included in each survey. This gave a response rate of 94.4% (n = 19,207/20,339). The remaining 5.6% (1,132 of 20,339) had missing data on different household variables and were not included in the analysis. The households for which data were excluded were not different from other households in any aspect. The average family size was 5.1 (range 1-12). The mean age was 20.5 years, and the median age was 15 years. Table 1 shows the age distribution and other study characteristics.

**Table 1:** Socio-demographic, seasonal and residential characteristics of the study participants (N = 19,207), Butajira area, Ethiopia, 2008-2010

Variables	Total people Enrolled (N = 20339)*	Total examined in three altitudinal zones (N = 19207)		
	
		Low (N = 5548)	Mid-level (N = 2040)	High (N = 11619)
**Age groups**				

0-11 months	330	106 (1.9)	31 (1.5)	171 (1.5)

12-23 months	553	156 (2.8)	44 (2.2)	323 (2.8)

2-4 years	2313	771 (13.9)	223 (10.9)	1219 (10.5)

5-9 years	3718	1079 (19.4)	377 (18.5)	2058 (17.7)

10-14 years	2880	782 (14.1)	329 (16.1)	1591 (13.7)

15-19 years	1993	493 (8.9)	224 (11.0)	1178 (10.1)

20-29 years	2666	747 (13.5)	242 (11.9)	1511 (13.0)

30-39 years	2652	685 (12.3)	237 (11.6)	1552 (13.4)

40-59 years	2360	536 (9.7)	234 (11.5)	1481 (12.7)

60-99 years	874	193 (3.5)	99 (4.9)	535 (4.6)

**Sex**				

Male	9873	2752 (49.6)	944 (46.3)	5655 (48.7)

Female	10466	2796 (50.4)	1096 (53.7)	5964 (51.3)

**Survey periods**				

21 Oct.-6 Nov.2008	3460	1003 (18.1)	353 (17.3)	2060 (17.7)

20 Jan.-4 Feb.2009	3336	928 (16.7)	348 (17.1)	1929 (16.6)

19 Jun.- 5 Jul.2009	3421	951 (17.1)	339 (16.6)	1937 (16.7)

18 Oct.-11 Nov.2009	3447	924 (16.7)	345 (16.9)	1941 (16.7)

27 Jan.-9 Feb.2010	3421	920 (16.6)	317 (15.5)	1890 (16.3)

5 Jun. -17 Jun. 2010	3254	822 (14.8)	338 (16.6)	1862 (16.0)

**Kebele**/Village				

**Hobe**	**3122**	**2943**	**-**	**-**

Dadesso	491	470 (8.5)	**-**	**-**

Horosso	1421	1382 (24.9)	**-**	**-**

Semeno	488	423 (7.6)	**-**	**-**

Wulbareg	722	668 (12.0)	**-**	**-**

**Bati Lejano**	**2817**	**2605**	**-**	**-**

Fasilo & Shibale	1062	928 (16.7)	**-**	**-**

Gobrano	444	367 (6.6)	**-**	**-**

Goyiban	1080	1080 (19.5)	**-**	**-**

Yimero	231	230 (4.1)	**-**	**-**

**Shershera Bido**	**3185**	**-**	**1568**	**1431**

Shershera	1658	-	1494 (73.2)	85 (0.7)

Bido	1527	-	74 (3.6)	1346 (11.6)

**Dirama**	**3462**	**-**	**472**	**2566**

Kelakel	3462	-	472 (23.1)	2566 (22.1)

**Wurib**	**3867**	**-**	**-**	**3771 (32.5)**

Wedo Zuriya	3867	-	-	3771 (32.5)

**Yeteker**	**3886**	**-**	**-**	**3851**

Agarega & Zenbaba Kolo	783	-		778 (6.7)

Kinbot and Jetena	1166	-	-	1150 (9.9)

Sunke & Wenz Akababi	1046	-	-	1032 (8.9)

Zenbaba Kolo & Shifo	891	-		891 (7.7)

*Numbers in () are percentages

### Community-based prevalence of malaria parasites

The overall prevalence of malaria was 0.93% [95% CI (0.79-1.07)] (178 malaria cases among 19,207 people). However, the prevalence varied among the villages, with the highest prevalence of 2.8% in Dadesso and Horosso villages (both below 1,850 masl), and the lowest prevalence (0.0%) at Sunke Wenz and Akababi village (2,100-2,180 masl) (data not presented). Malaria varied with altitude, with a prevalence of 1.91% [95% CI (1.55-2.27)] in low, 1.37% [95% CI (0.87-1.87)] in mid-level and 0.36% [95% CI (0.25-0.47)] in high altitude zones. The highest prevalence was found at low altitude between October and November 2009 (Table 2).

**Table 2:** Prevalence of malaria in different altitudes in six survey periods, Butajira area, Ethiopia, 2008-2010

	Low		Mid-level		High		
		
Survey period	Positive/Total sampled	Prevalence,%	Positive/Total sampled	Prevalence, %	Positive/Total sampled	Prevalence, %	Total, n (%)
21 Oct.-6 Nov.2008	16/1003	1.59	2/353	0.57	2/2060	0.97	20 (11.36)

20 Jan.-4 Feb.2009	6/928	0.65	2/348	0.57	3/1929	0.15	11 (6.25)

19 Jun.- 5 Jul.2009	16/951	1.68	8/339	2.36	3/1937	0.15	27 (15.34)

18 Oct.-11 Nov.2009	43/924	4.76	8/345	2.32	20/1941	1.03	71 (40.34)

27 Jan.-9 Feb.2010	21/920	2.28	6/317	1.89	7/1890	0.37	34 (19.32)

5 Jun. -17 Jun. 2010	4/822	0.49	2/338	0.59	7/1862	0.37	13 (7.39)

Total	106/5548	1.91	28/2040	1.37	42/11619	0.36	176 (100)*

*Two mixed infections of *Plasmodium falciparum *and *Plasmodium vivax *were not included

**Figure 2:**
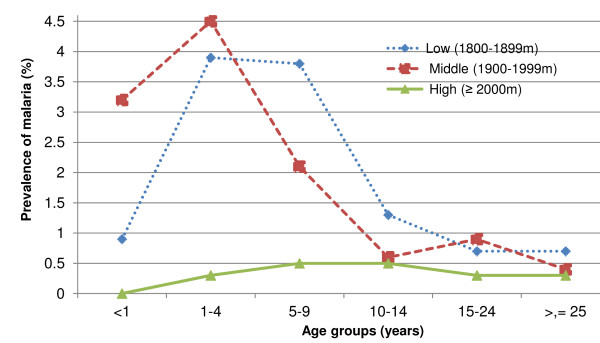
**Age-specific prevalence of all forms of malaria infection by altitudinal zone, Butajira area, Ethiopia, 2008-2010**.

Two persons with *P. vivax*-positive slides, one from the lowlands and one from the highlands, reported that they had travelled outside their villages. Malaria infection varied among age groups, and in a different way at varying altitudes. At mid-level altitudes, malaria infection reached its peak in children aged one to four years, and at low altitude in children aged one to nine years. However, malaria prevalence at higher altitude was low and was similar across all age groups (Figure 2). Table 3 shows that *Plasmodium falciparum *malaria occurred rarely throughout the survey periods, with relatively more cases during the survey performed in October-November 2009 in the low altitude zone. *Plasmodium vivax *was found in all survey periods; however, the prevalence of *Plasmodium vivax *differed with respect to survey period and altitude (Table 4).

**Table 3:** Prevalence of *Plasmodium falciparum *(n = 22) in Butajira area, Ethiopia, 2008-2010

Altitude Zone	Low		Middle		High	
**Survey period**	**Positive/Total sampled, n**	**Prevalence, %**	**Positive/Total sampled, n**	**Prevalence, %**	**Positive/Total sampled, n**	**Prevalence, %**

21 Oct.-6 Nov.2008	0/1003	0	2/353	0.57	0/2060	0

20 Jan.-4 Feb.2009	0/928	0	0/348	0	0/1929	0

19 Jun.- 5 Jul.2009	6/951	0.63	0/339	0	0/1937	0

18 Oct.-11 Nov.2009	11/924	1.19	0/345	0	3/1941	0.15

27 Jan.-9 Feb.2010	0/920	0	0/317	0	0/1890	0

5 Jun. -17 Jun. 2010	0/822	0	0/338	0	0/1862	0

Total	17/5548	0.31	2/2040	0.10	3/11619	0.03

**Table 4:** Prevalence of *Plasmodium vivax *(n = 154) in Butajira area, Ethiopia, 2008- 2010

Altitude Zone	Low		Middle		High	
**Survey period**	**Positive/Total sampled, n**	**Prevalence, %**	**Positive/Total sampled, n**	**Prevalence, %**	**Positive/Total sampled, n**	**Prevalence %**,

21 Oct.-6 Nov.2008	16/1003	1.59	0/353	0	2/2060	0.97

20 Jan.-4 Feb.2009	6/928	0.65	2/348	0.57	3/1929	0.15

19 Jun.- 5 Jul.2009	10/951	1.05	8/339	2.36	3/1937	0.15

18 Oct.-11 Nov.2009	32/924	3.46	8/345	2.32	17/1941	0.87

27 Jan.-9 Feb.2010	21/920	2.28	6/317	1.89	7/1890	0.37

5 Jun. -17 Jun. 2010	4/822	0.49	2/338	0.59	7/1862	0.37

Total	89/5548	1.60	26/2040	1.27	39/11619	0.34

## Discussion

This study shows that malaria occurs sporadically at high altitudes, and that the prevalence of malaria increases towards the lowlands. Malaria occurs throughout the year, but predominantly after the main rainy season. However, the prevalence varied greatly among villages at all altitudes. More children had malaria in the lowlands than in the highlands, which suggest that the highland population has lower immunity to malaria, as a result of limited prior exposure to the disease.

The strengths of the present study lay in the use of representative samples, the people investigated being residents of the study area, and the assessment of malaria in different seasons repeatedly for two consecutive years. Thus, this study provides an unbiased estimate of malaria prevalence in a localized area with increased epidemic risk.

The finding of malaria cases at high altitudes is consistent with a study performed in a nearby area [20]. However, that study may have overestimated the prevalence because it was performed only in the peak season for malaria transmission. Another study reported malaria infection at altitudes as high as 2,500 masl, and even extending above 3,000 masl [21]. However, the geographic origin of the cases was not well documented.

The low estimate for malaria prevalence obtained in this study is in agreement with another study carried out at a similar altitude in Ethiopia [22], and a previous estimate obtained in another region with unstable malaria transmission [23]. Areas of such low endemicity may be categorized as hypoendemic [24,25], and they are considered to be vulnerable to epidemics.

The variability in the prevalence of malaria among villages is in agreement with previous studies in highland areas of Ethiopia [26], and studies performed in other regions with similar unstable malaria transmission [23,27,28]. This can be explained by the clustering of mosquitoes in villages near to mosquito breeding sites. Although this study lacks mosquito-sampling data, the influence of the clustering of mosquitoes in space on similar malaria case distributions is well documented elsewhere [29]. One study found a higher density of *An*. *arabiensis *in parts of a village with higher malaria prevalence near to Lake Ziway in Ethiopia [10]. Therefore, identification of areas in which malaria cases are clustered in space could be helpful in the design of focused interventions against malaria.

The present results showed the seasonality of malaria, and this is in agreement with other studies [20,26]. The occurrence of malaria depends on adequate rainfall and temperature. In areas with a temperate climate, transmission of malaria is commonly limited to months in which the average temperature is above the minimum required for sporogony [24]. This explains the inverse relationship of decreasing malaria prevalence with increasing altitude. Temperature is a limiting factor for sporogony of the malaria parasite. A previous study demonstrated that an increase in altitude played a protective role against malaria infection [21]. The finding of malaria at high altitude should alert local health authorities to target malaria case detection and improve surveillance in highland areas, including those above 2,000 masl. The present finding of endemic malaria at altitudes as high as 2,200 masl during a non-epidemic year might be interpreted as the emergence of endemic malaria in the highlands. An increase in the daily minimum temperature of 0.4°C per decade has been recorded in the highlands of Ethiopia [30]. Presumably, this temperature rise has favoured the occurrence of malaria by keeping minimum temperatures in the highlands above the threshold for development of the malaria parasite in mosquitoes [24].

The finding of an increased proportion of *vivax *malaria is consistent with a study at high altitude in the Butajira area [20], and a study in the Akaki area [13]. Recent studies also show a shift from falciparum to vivax malaria [26,31]. However, other studies report the consistent dominance of *Plasmodium falciparum *[32-34].

The increase in *vivax *malaria in highland-fringe areas could be explained by the high transmissibility of *P. vivax*, which is related to its typical biological features, including the immediate appearance of gametocytes, the presence of hypnozoites, and shorter sporogony [24,25,35]. A more than two-fold increase in the rate of drug resistance has been reported in south-central Ethiopia, close to the study area [36]. Thus chloroquine-resistant *vivax *malaria may have accounted for the dominance of *vivax *observed in this study. Some authors also speculate that the intensive malaria control efforts in Ethiopia, which have been implemented since 2005 [37], could explain the dominance of *Plasmodium vivax *[31].

## Competing interests

The authors declare that they have no competing interests.

## Authors' contributions

AW contributed to conception and design, acquisition of data, analysis and interpretation of data, and drafting the manuscript. WD contributed to conception and design of the study and reviewed the manuscript. AA contributed to conception and design of the study and reviewed the manuscript. BL contributed to conception and design, analysis and interpretation of data, and reviewed the manuscript. BL, AA, WD and AW reviewed the paper and all authors approved the final version.
